# Surface ocean microbiota determine cloud precursors

**DOI:** 10.1038/s41598-020-78097-5

**Published:** 2021-01-11

**Authors:** Karine Sellegri, Alessia Nicosia, Evelyn Freney, Julia Uitz, Melilotus Thyssen, Gérald Grégori, Anja Engel, Birthe Zäncker, Nils Haëntjens, Sébastien Mas, David Picard, Alexia Saint-Macary, Maija Peltola, Clémence Rose, Jonathan Trueblood, Dominique Lefevre, Barbara D’Anna, Karine Desboeufs, Nicholas Meskhidze, Cécile Guieu, Cliff S. Law

**Affiliations:** 1 Laboratoire de Météorologie Physique (LaMP), Université Clermont Auvergne, CNRS, 63000 Clermont-Ferrand, France; 2 Laboratoire d’Océanographie de Villefranche (LOV), Sorbonne Université, CNRS, 06230 Villefranche-sur-Mer, France; 3 Mediterranean Institute of Oceanography UM110, Aix-Marseille University, Toulon University, CNRS, IRD, 13288 Marseille, France; 4 GEOMAR, Helmholtz Centre for Ocean Research, 24105 Kiel, Germany; 5 School of Marine Sciences, University of Maine, Orono, ME 04469 USA; 6 MEDIMEER, UMS3282 OSU OREME, Université de Montpellier, CNRS, IRD, Sète, France; 7 National Institute of Water and Atmospheric Research (NIWA), Wellington, New Zealand; 8 Department of Marine Sciences, University of Otago, Dunedin, New Zealand; 9 Laboratoire Chimie Environnement (LCE), UMR 7673 CNRS, Université Aix-Marseille, 13331 Marseille, France; 10 LISA, UMR CNRS 7583, Institut Pierre Simon Laplace (IPSL), Université de Paris, Université Paris-Est-Créteil, Créteil, France; 11 North Carolina State University, Raleigh, USA

**Keywords:** Atmospheric science, Marine biology

## Abstract

One pathway by which the oceans influence climate is via the emission of sea spray that may subsequently influence cloud properties. Sea spray emissions are known to be dependent on atmospheric and oceanic physicochemical parameters, but the potential role of ocean biology on sea spray fluxes remains poorly characterized. Here we show a consistent significant relationship between seawater nanophytoplankton cell abundances and sea-spray derived Cloud Condensation Nuclei (CCN) number fluxes, generated using water from three different oceanic regions. This sensitivity of CCN number fluxes to ocean biology is currently unaccounted for in climate models yet our measurements indicate that it influences fluxes by more than one order of magnitude over the range of phytoplankton investigated.

## Introduction

The natural variability of sea spray aerosol (SSA) number fluxes and chemical composition is poorly understood, yet it has been shown to play a major role in the Earth climate system^1^, largely through their effect on maritime cloud properties. Cloud radiative properties depend on cloud droplet number concentrations (CDNC)^2,3^, which are a function of atmospheric particle concentrations that serve as cloud condensation nuclei (CCN). In the Southern Ocean, a region with limited anthropogenic aerosols, SSA may contribute a significant fraction of the CCN^4^ leading to elevated CDNCs in low-level maritime boundary layer clouds^5^. Despite elevated CDNCs in the Southern Ocean being associated with regions of high Chlorophyll-a (Chl-a) concentration^6,7^, the processes linking marine biota, marine aerosols, and CDNC are still not well understood and remain a topic of a three-decades long debate^8^. Although the biogenic origin of organic matter in sea spray has been extensively studied^9–13^, empirical evidence that marine biota modifies the sea spray number concentration flux to the atmosphere is scarce^14^ and yet to be formalized. Laboratory studies have shown an increase of SSA number concentrations related to organic exudates, phytoplankton and/or bacteria^15,16^ and some in-situ studies have suggested that SSA number concentration and organic content are related^17–19^. An elevated sea spray number production was observed for SSA generated from biologically productive seawaters relative to oligotrophic waters^15–20^. However, to our knowledge, a quantitative link between phytoplankton groups and SSA number fluxes, a critical component for simulating the impact of marine aerosols on CCN number concentrations^21^ and climate^22^, has not been established.

### Relevant sea spray emission properties for cloud condensation nuclei fluxes

**Figure 1:**
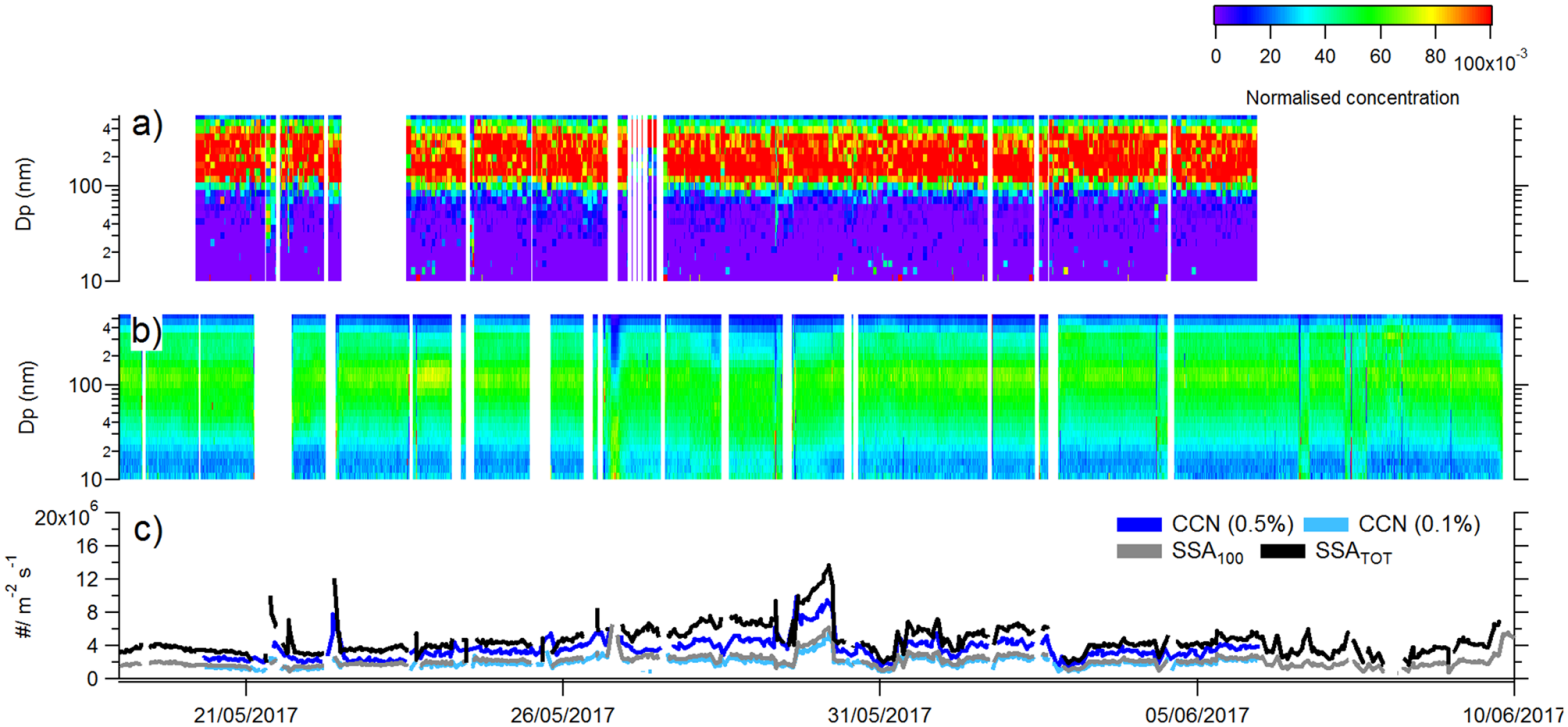
Time series along the PEACETIME (ProcEss studies at the Air-sEa Interface after dust deposition in The Mediterranean sea) ship track of **(a)** CCN_0.1%_ concentrations normalized to the sea spray concentration at given particle diameter (Dp). The activation diameter of SSA into cloud droplet at 0.1% supersaturation is visible at the change of colour from purple to red **(b)** sea spray concentration at given diameters (Dp) normalized with the total sea spray concentration, **(c)** flux of sea spray aerosol number (SSA_TOT_), flux of sea spray larger than 100 nm number (SSA_100_), and flux of CCN at 0.5% and 0.1% supersaturation, CCN_0.5%_ and CCN_0.1%_, respectively.

CCN number fluxes measured from a continuous flow-through underway seawater plunging jet system^13^ varied by a factor of 3 along the ship track in the Mediterranean Sea (Fig. 1c). The variability of the CCN concentration may depend on the variability in the available SSA hygroscopicity, size, and number concentration, each of which was tested. According to Fig. 1a, the activation diameter of sea spray at 0.1% supersaturation is stable at ~ 100 nm, indicating very little changes in hygroscopicity of SSA. Hence, changes in the hygroscopic properties of SSA alone were insufficient to explain the observed spatial and temporal variation of the CCN number fluxes over different regions of the Mediterranean Sea. The average size distribution of SSA was dominated by 115 nm particles (see lognormal mode characteristics in the Supplementary Information (SI)) and showed little variation along the ship’s track as well (Fig. 1b), consistent with previous experiments in the Mediterranean Sea ^13,23^. This indicates that changes in the size of SSA were not able to explain the variation in CCN production fluxes neither. It can be seen in Fig. 1c that the variations in the CCN number fluxes closely follow the variations in the SSA number flux. The cloud condensation nuclei flux at 0.1% supersaturation (CCN_0.1%_ flux) can be predicted with a mean 13% accuracy from the number flux of sea spray particles greater than 100 nm (SSA_100_ flux) (CCN_0.1%_ = 0.78 * SSA_100_ + 72; R = 0,88), independently of the SSA size and hygroscopicity. As there are more data available for SSA number fluxes than CCN number fluxes, we will in the following discussion focus on the environmental factors influencing the SSA_100_ flux, assumed to control the CCN_0.1%_ flux to the atmosphere.

### Seawater properties determine sea spray number fluxes

**Figure 2:**
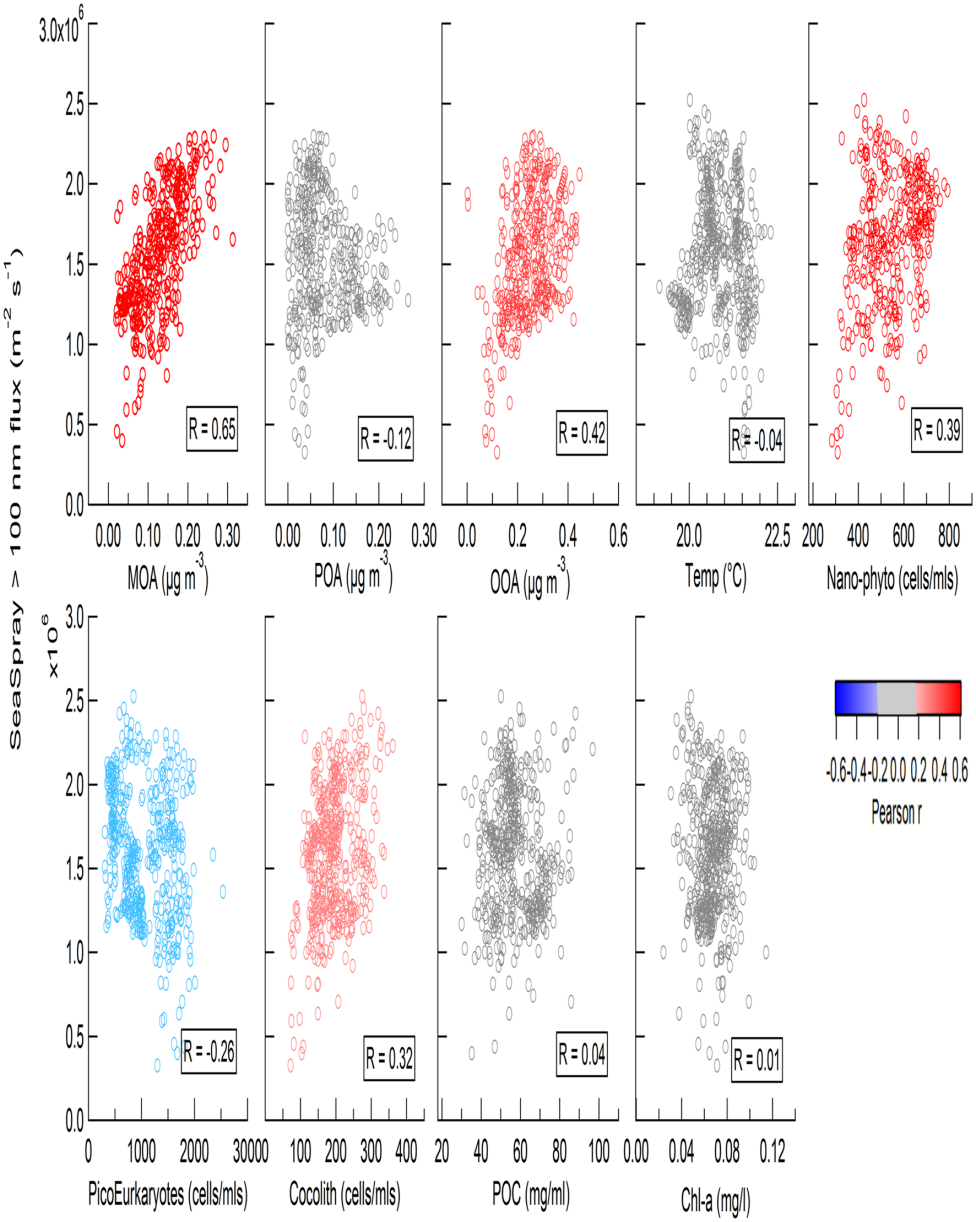
Correlation plots between the sea spray larger than 100 nm number fluxes (m^−2^ cm^−1^) and the three classes of organic matter in nascent sea spray (MOA, POA, OOA), Sea Surface Temperature (SST, °C), seawater cell abundances (cells ml^−1^) for nanoeukaryotes (2–20 µm size; *NanoPhyto*), picoplankton (3 µm or less in size), coccolithophore-like cells, particulate organic carbon (POC, mg ml^-1^), and total surface Chl-*a* (µg l^−1^), from the Mediterranean cruise data set. Red datapoints indicate a significant positive correlation, Blue a significant negative correlation and grey no significant relationship at the 99.9% confidence level.

CCN number and CDNC have only been tentatively, but not consistently, related to seawater Chl-*a* concentration in the literature, indicating that Chl-*a* is not an optimal proxy for predicting marine CCN fluxes. In the Mediterranean Sea, the Chl-*a* spatial variation did not match the sea spray originating CCN spatial variation (Fig. S2a,b). Amongst the other seawater biological parameters measured (including nanoeukaryotes, picoeukaryotes, coccolithophores, cryptophytes and Synechoccocus cell abundances), the strongest linear correlation was observed between SSA_100_ and nanoeukaryotic cell abundance (NanoPhyto) (R = 0.39, p < 0.001) (Fig. 2). We further examined the relationship between SSA_100_ and the nanophytoplankton cell abundance in other data sets.

**Figure 3:**
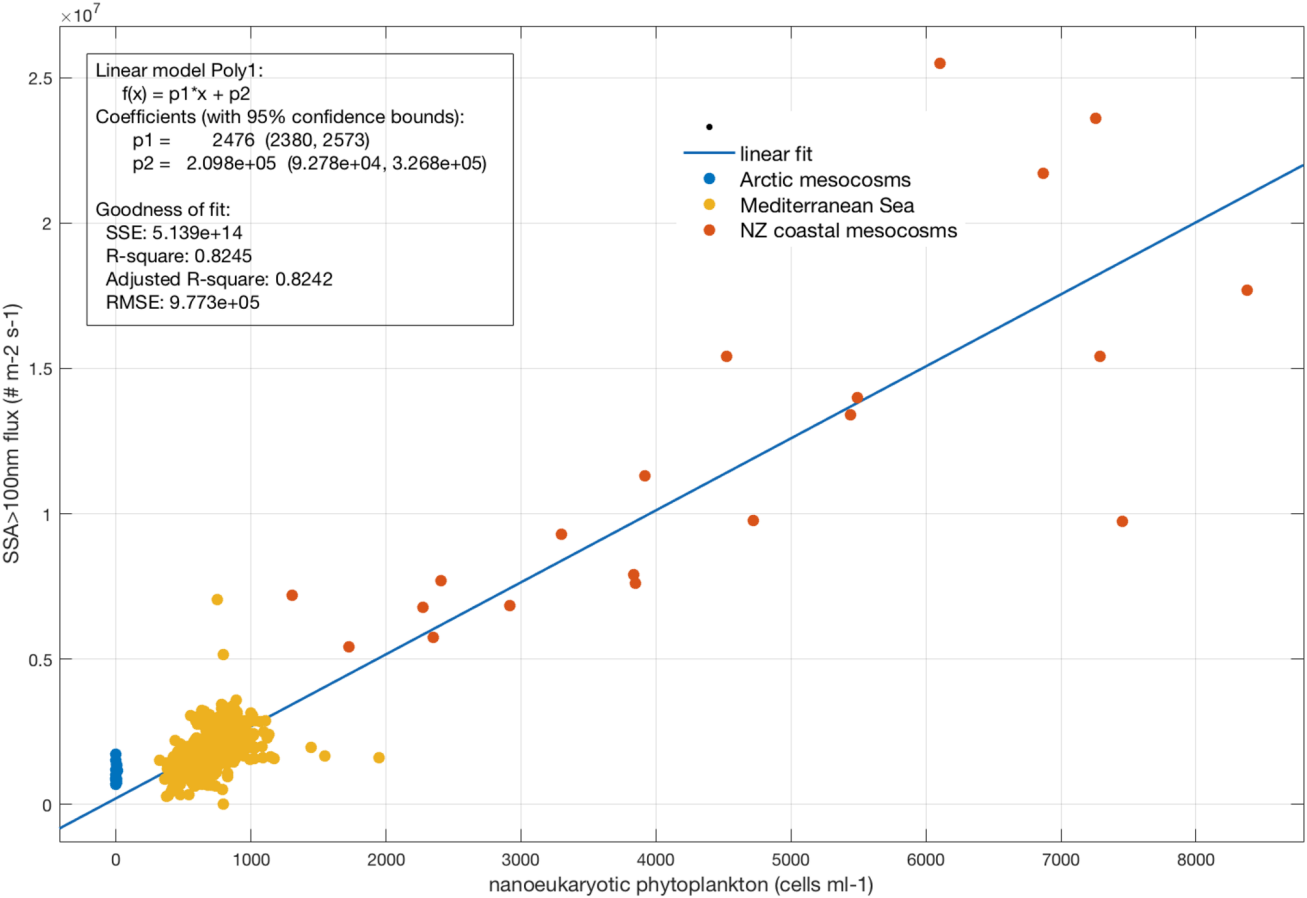
SSA larger than 100 nm flux number F_SSA100_ (# m^−2^ s^−1^) calculated for a wind speed of 9 m s^−1^ and 15 °C sea temperature as a function of the NanoPhyto (cells ml^−1^) from different regions. The solid line indicates the linear fit to the data set.

Similar SSA generation experiments were performed using South West Pacific (NZ coastal) and Arctic coastal waters at different periods of the year. The NZ coastal waters were sampled during the austral spring when productivity was elevated. When relating the SSA_100_ production flux (F_CN100_) from the NZ coastal waters to biogeochemical seawater properties, the highest correlations were again observed with NanoPhyto cell abundances, with similar coefficients to that of Mediterranean oligotrophic waters (Table 1). To account for the temperature dependence of SSA number production fluxes ^26,27^ in the different data sets, all fluxes were normalized to a 15 °C SST, following Salter et al. (2015). The merged data sets provided a single linear relationship between F_SSA100_ and NanoPhyto (Fig. 3) with a greater statistical significance (higher R value) than the individual regional data sets (Table 1). Using the resulting linear relationship between F_SSA100_ and NanoPhyto for the merged data set, we define the F_SSA100_ in biologically-free seawaters, i.e. containing only inorganic chemical components (F_SSA100INORG_) as the intercept of the y axis (2.1 × 10^5^ m^−2^ s^−1^). We then express F_SSA100_ as a function of F_SSA100INORG_ in Eq. (1) for a larger use of our parameterization in regional and global models.

1 $$ {\text{F}}_{{{\text{SSA1}}00}} = \, ({1}.{18} \times {1}0^{{ - {2}}} *{\text{ NanoPhyto }} + {1})*{\text{F}}_{{{\text{SSA1}}00{\text{INORG}}}} $$

**Table 1:** Fitting parameters (a and b) and correlation coefficients (Pearson R) for the linear relationship between the production flux of SSA100 (F_SSA100_, m^−2^ s^−1^) and eukaryotic nanophytoplankton cell abundance (NanoPhyto, in cells ml^-1^) in the form of F_SSA100_ = a * NanoPhyto + b for a wind speed of 9 m s^-1^ and temperature of 15 °C. The correlation coefficient Spearman Rho between these two variables is also indicated. Average, standard deviation and range (10–90 percentiles) of Chl-*a* and NanoPhyto for the three measurement campaigns.

Seawater geographical origin	a	b	R	Rho	P value	Chl-a (μg l^−1^)	NanoPhyto (cells ml^-1^)
Mediterranean	2.17 × 10^3^	3.83 × 10^5^	0.72	0.51	< .00001	0.03 ± 0.01 0.02–0.04	546 ± 148 390–703
South west Pacific	2.20 × 10^3^	2.04 × 10^6^	0.77	0.85	< .00001	2.13 ± 0.40 1.65–2.60	4880 ± 2390 1840–8250
Arctic	–	–	–		–	0.01–0.3	4.2 ± 4.3 0–9.4
Merged Mediterranean, NZ coastal and Arctic	2.48 × 10^3^	2.10 × 10^5^	0.90	0,60	< .00001	0.01–2.60	0–8250

Although the NZ coastal data set shows a large NanoPhyto range and the F_SSA100_–NanoPhyto relationship is similar to that of the Mediterranean dataset, combining all data sets provides the broadest coverage of F_SSA100_ and NanoPhyto. Therefore, we suggest that this correlation, derived from a two-orders of magnitude range of NanoPhyto cell abundances, could, as a first approximation, be applicable to the spatial range and trophic diversity across the global oceans. However, while NanoPhyto were almost absent in Arctic seawater during early springtime (Table 1), the average F_SSA100_ obtained for this data set (1.1 ×10^6^ m^−2^ s^−1^), although close to F_SSA100INORG_, still differs by a factor of 5. This lack of agreement suggests that a different parameter is controlling F_SSA100_ in the Arctic mesocosm experiment. This illustrates the need for a better mechanistic understanding of the factors driving the relationship between NanoPhyto and F_SSA100_.

### Potential mechanisms for a biologically driven sea spray number fluxes

We examined relationships between SSA_100_ and organic content of SSA. In the Mediterranean dataset, three main groups of organic species with different oxidation levels were identified in SSA using a positive matrix factorization: a primary-like OA group (POA), an oxidized organic group (OOA), and a mixed (primary and oxidized) group (MOA)^25^. As shown in Fig. 2, SSA_100_ showed the strongest correlation with MOA (R = 0.65, p < 0.001) with a higher significance than for the correlation between NanoPhyto and SSA_100_ (R = 0.39, p < 0.001); this infers that NanoPhyto influence SSA_100_ by producing MOA but may not be the only source of MOA. The organic mass fraction of submicron nascent sea spray did not vary significantly along the cruise track (22 ± 6%) and the contribution of MOA to total sea spray mass is less than 2%. Therefore, the hypothesis that organics simply added to the inorganic sea salt flux cannot explain the observed F_SSA100_ increase by a factor 3 with MOA.

The most plausible mechanism is that F_SSA100_ is influenced by the presence of organics surfactants in the seawater and surface microlayer (SML)^28^ which influence bubble bursting. This hypothesis is based on the fact that the SML is considered to be formed very rapidly (on the order of less than 20 s) via bubbling^29^, and hence a SML would have been present in the sea spray generator. Organic surfactants may have different effects on the stability of bubbles. Soluble surfactants lead to higher bubble lifetimes (foam stabilization effect), making bubbles films thinner and hence decreasing the SSA number fluxes to the atmosphere^30,31^. On the contrary, the presence of fatty acids suppresses foam formation, leading to shorter bubble lifetimes and hence thicker bubble films with the effect of increasing SSA number fluxes^32,33^. MOA concentrations measured in the Mediterranean SSA were correlated with the enrichment of DOC in the SML relative to the underlying surface seawater^28^, suggesting that MOA is itself enriched in the SML due to surface active properties. The MOA mass spectrum in the Mediterranean SSA contained mixed signatures of amino acids and fatty acids that were potentially responsible for the observed decrease in surface tension^25^. In the NZ coastal seawater, surface tension decreased with increasing seawater particulate saturated fatty acid concentration (R = 0.66, p < 0.01). In the Mediterranean data set, we also found that SSA_100_ is correlated with Transparent Exopolymer Particles (TEP) abundance (R = 0.46, p < 0.01), which are microgels that are produced by coagulation of phytoplankton exudates and also have surface active properties^34^. The correlations between SSA and the different organic tracers measured during the different campaigns point to the presence of fatty acids combined with other organics, originating from exudates, in shaping the sea spray fluxes. In line with this hypothesis, past studies have shown a synergistic effect of surfactant mixtures on SSA fluxes compared to single component monolayers^33^ and lipopolycarbonates were found to be a major fraction of marine primary organic matter^35^. Particulate fatty acids are the main components of lipids in cell membranes, therefore they are of primary origin, and the lack of a clear diurnal variation of SSA_100_ (Fig. S2a), suggests that the SSA flux variability is not a result of aqueous-phase photochemistry. The absence of a temporal shift between high NanoPhyto and high F_SSA_ indicates that if organics are released by cell lysis, as suggested by earlier studies^12,36,37^ the process is continuous.

We have made a step towards a mechanistic understanding of the processes responsible for the increase of the SSA number fluxes using seawater biology, and we propose that this mechanism is primarily the influence of organic matter on bubble lifetime; however, more work is required to further ascertain this hypothesis. The relationship between nanoeukaryotes and MOA, and particularly whether certain species are responsible for the release of surface active compounds, requires further research. In the NZ coastal mesocosms, the small pennate diatom species *Nitzchia closterium* represented a significant fraction of the nanophytoplankton, and was also significantly correlated to SSA_100_ number concentration (R = 0.70, p < 0.001), but we do not have species information for the NanoPhyto in the other two datasets. However, establishing these relationships in natural marine systems is a first step towards identification of the mechanistic processes that will stimulate further research.

### Impact on the variability of CCN fluxes driven by the marine biota and future implications

**Figure 4:**
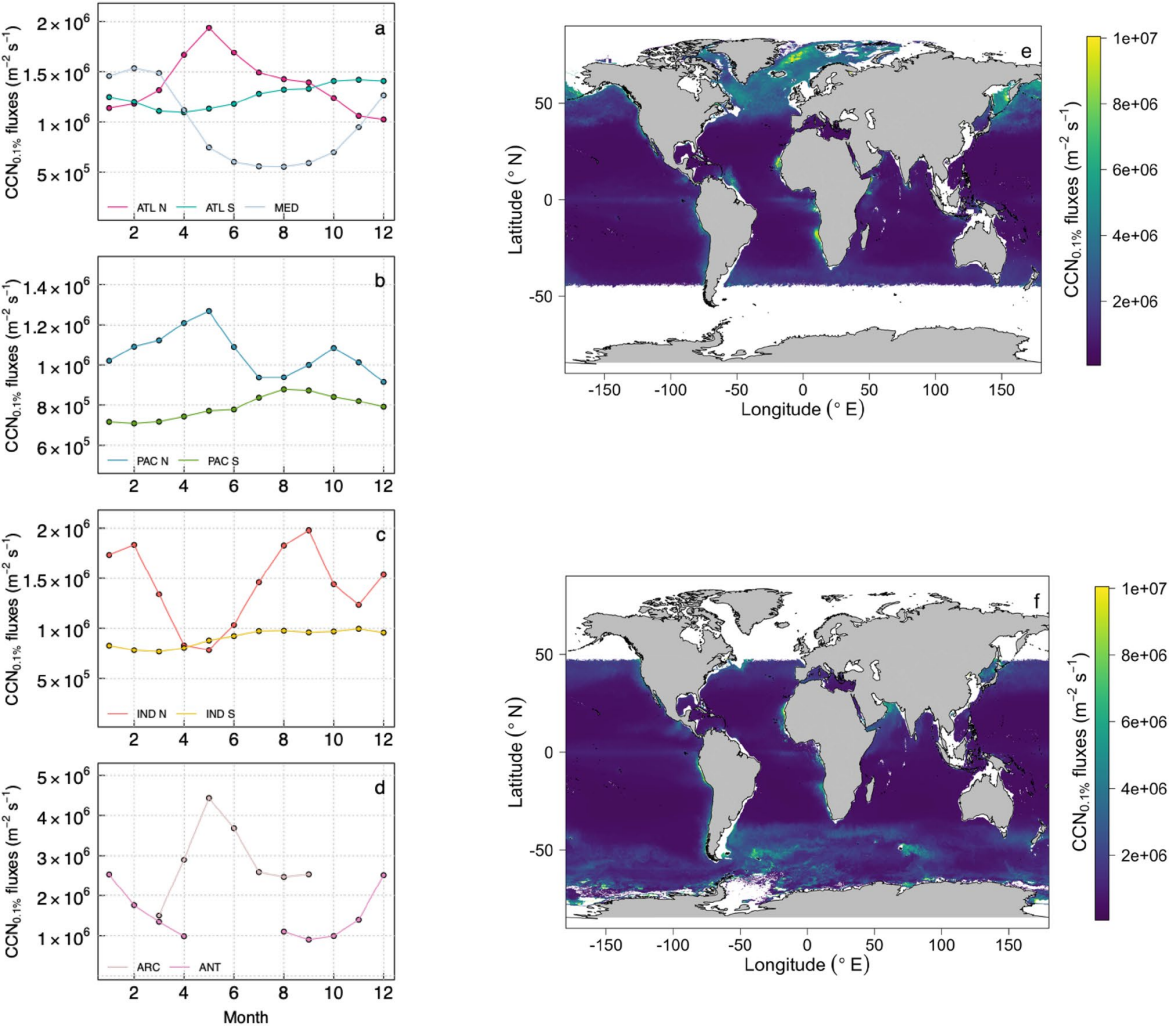
Global CCN_0,1%_ emission fluxes (m^−2^ s^−1^) computed for a 15 °C temperature from satellite-based nanoplankton cell abundance in June **(e)** and December **(f)**. CCN fluxes were computed using Eq. (1), and assuming that CCN_0,1%_ is equal to SSA_100_. Median annual trends are represented for the different oceanic basins **(a)** ATL N: North Atlantic, ATL S: South Atlantic, MED: Mediterranean Sea; **(b)** PAC N: North Pacific, PAC N (South Pacific), **(c)** IND N (North Indian Ocean), IND S (South Indian Ocean), **(d)** ARC (Arctic Ocean), ANT (Southern Ocean). Maps provided **(e,f)** were created using R version 3.4.2. (https://www.R-project.org/).

The variability of CCN_0.1%_ number fluxes at the global scale resulting from the spatial and temporal variations of NanoPhyto was computed using the ocean colour satellite-based phytoplankton group retrieval following Uitz et al. ^38^ and the parameterization given Eq. (1), with the assumption that all SSA_100_ will activate at 0.1% supersaturation. During summer, the resulting CCN_0.1%_ number fluxes are a factor of 5 higher at high latitudes compared to the subtropical areas in a given hemisphere (Fig. 4). Fluxes show little seasonal variation in the subtropical and tropical zones but can vary by at least a factor of 2 between winter and summer at high latitudes (Fig. 4). Currently, the impact of surface ocean biogeochemistry is only considered in global models by the addition of simulated marine organic matter to the CCN mass, which is predicted to increase CCN number concentration by < 50%^39^. From our results, the biological impact on the CCN flux number via its role in the bubble bursting process may be much higher (by up to a factor 5) than this 50% increase.

## Methods

### Field campaigns description

In the frame of the PEACETIME (MERMEX CHARMEX) project, an oceanographic cruise (https://doi.org/10.17600/17000300) onboard the R/V ‘Pourquoi Pas?’ took place in the Western/Central Mediterranean Sea May 10–June 11, 2017. The Mediterranean Sea was typical of oligotrophic conditions throughout the transect characterized by shallow mixed layer depth, low nutrients and chlorophyll-a concentrations. A clean pumping system enabled continuous sampling of seawater at 5 m depth and transfer to a sea spray generation device. In parallel, a number of parameters characterized surface water during the whole transect (2800 nautical miles).

The ME3 campaign was organized in New Zealand coastal waters (Evans Bay) in November 2017. The experimental setup consisted of three mesocosms containing 4200 l of seawater and 170 l headspace flushed with filtered air. Nutrients were added to all mesocosms daily over 21 days. One mesocosm served as control while one mimicked conditions of year 2100 (temperature increased by 2.6 °C and pH lowered by 0.5) and one mimicked conditions of year 2150 (temperature increased by 4.5 °C and pH lowered by 0.33. A total of 3.4 L of water collected from each mesocosm at 2 m depth from a well-mixed water column was used daily to generate sea spray as described below.

The Arctic mesocosm experiment was carried out in the framework of the MACA project (Marine Aerosol impact on Clouds in the Arctic) from the 5th to the 22nd of March 2017 in the Kongsfjorden next to the Ny-Alesund research station (78◦55′42.5" N 11◦56′13.3" E), Svalbard. The goal of the MACA project was to evaluate the impact of pollution on marine aerosol emission and cloud-related properties. Mesocosms enclosed 2260 L of seawater and 1 m^3^ of natural particle filtered air in a UV-transparent enclosure. Three mesocosms were used, one as a control, and the two others seeded with ammonium sulfate and ammonium nitrate rich rainwater, at concentrations typical of an average rain occurring in the area, and three times this average rain water concentration. Mesocosms were sampled daily from the 5th of March to the 22nd of March, with the exception during the 10th and the 11th due to bad weather conditions. Collection of samples were performed in the early morning using 20 L polycarbonate carboys, cleaned with ultrapure water at 1 m depth in well mixed water columns. A total of 3.4 L of water collected from each mesocosm was used daily to generate sea spray as described below.

### Nascent sea spray generation and characterization

The same sea spray generation device was used in all three campaigns (PEACETIME, ME3, MACA), and was previously described in Schwier et al. (2015, 2017)^13,24^. Experimental procedures used to generate nascent sea spray particles were also similar in all experiments, with the exception that the sea spray in the Mediterranean was generated continuously, while the sea sprays from NZ-coastal and Arctic mesocosms were generated as discrete daily samples. Briefly, the sea spray generator consists of a glass tank—20 × 20 cm and 25 cm high- which is filled with 3.6 L of seawater. In the remaining volume, particle-free air is flushed to maintain a constant over-pressure. The air flush, which was kept constant over the course of each measurement campaign, was similar for all three experiments (Mediterranean, Arctic, NZ coastal) and served to reproduce the wind and ensure the absence of ambient room particles. Inside the sea spray generator, seawater was forced through eight 1 mm nozzles and form vertical plunging jets. The plunging jet creates an air entrainment that reproduces the bubble size distribution naturally equivalent to the bubble size distribution of natural wave breaking^40,41^. The water flow rate ranged from 1.6 to 1.8 L min^−1^ depending on the experiment but was kept constant throughout each experiment. During PEACETIME, the system was used with seawater circulating in an open loop, continuously circulating underway seawater with a peristaltic pump. This type of sea spray generator was shown to successfully reproduce the average size distribution shape of ambient aerosols sampled in clean oceanic sector^40,41^. Upscaling of the fluxes obtained using the plunging jets system to ambient wind dependent SSA fluxes is described in the production flux calculation section. The temperature of the seawater present in the sea spray generator varied according to the experiment. For the PEACETIME dataset, SST followed the underway SST and stayed fairly constant at 20.4 ± 1.2 °C. For the Arctic and NZ coastal water experiments, SSW temperature during sea spray generation were 13.4 ± 1.9 °C and 17.7 ± 2.2 °C respectively.

A CCNc-DMPS instrument was connected to the sea spray generator for measuring the sea spray number size distribution, and CCN number size distribution simultaneously. The custom-made instrument consists of a 2.5 micron impactor preventing large sea spray from entering the instrument, an aerosol neutralizer, a differential mobility Analyzer (DMA) column to select sea spray per size, followed by a Condensation Particle Counter (CPC) and a miniature continuous-flow stream-wise thermal-gradient CCN chamber (CCNc) in parallel. A CPC TSI 3010 was used in parallel to the DMPS, for quality check of the DMPS size distributions. All aerosol properties were measured after the aerosol was dried through a 1 m diffusion dryer after the sea spray generator, leading to < 40% RH in the instruments. Activation diameters at which sea spray activates to cloud droplets were calculated as described in Asmi et al. (2012)^42^. The chemical composition of sea spray was obtained using a Time of Flight Aerosol Chemical Speciation Monitor (ToF-ACSM^43^), on-line from the sea spray generator for the PEACETIME campaign. The treatment of data from the ACSM instrument and the subsequent positive matrix factorization of the measured organic aerosol^25^. A brief description will be provided here. Data acquired with the ToF-ACSM was analysed using ToFWARE v2.6.11. Raw aerosol mass spectra were treated with a modified version of the standard fragmentation table^44^. Since the ACSM instrument was sampling seawater directly the contribution of seasalt related aerosols was high (although the instrument is not designed to provide quantitative measurements of refractory material). In order to extract these seasalt related species and to avoid them dominating the organic aerosol spectra they were removed and classified into a seasalt related aerosol. This classification included ion signals at mz 23 (Na^+^), mz 58, mz 60 (NaCl), mz 81, mz 83 (NaCl)Na^+^^45^. Positive matrix factorization was then performed using the PMF evaluation tool v2.04. Organic related mz (excluding those possibility linked with NaCl) from 0 to 200 amu were included in the analysis. The temporal variability in the organic signal was low, making it difficult to extract different sources of organic species.

### Production fluxes calculations

The sea spray number flux F_CN_ (# m^−2^ s^−1^) was calculated from the SSA concentration (#m^−3^), flushing air flow (m^3^ s^−1^) and surface of seawater in the sea spray generator (m^2^). F_CN_ can also be expressed as a function of the volume of air entrained in the seawater by the plunging jets system F_ent_ (m^3^ m^−2^ s^−1^) following Eq. (2):

2 $$ {\text{F}}_{{{\text{CN}}}} \left( {\# {\text{ m}}^{{ - {2}}} {\text{s}}^{{ - {1}}} } \right) = {\text{ p F}}_{{{\text{Ent}}}} \left( {{\text{m}}^{{3}} {\text{m}}^{{ - {2}}} {\text{s}}^{{ - {1}}} } \right) $$

where p is the production efficiency per volume of air entrained, and F_ent_ the volume of air entrained. F_ent_ was measured as a function of seawater flow rate from our plunging jet system using an experimental setting that isolates a single jet from its environment equipped with an online air flowmeter, in a similar manner to that described by Salter et al. (2014)^46^. For relating the fluxes obtained from the plunging jet system to a wind speed-related flux in the ambient atmosphere, we followed the approach from Long et al. (2011)^47^. The authors calculate that the volume of air entrained in a plunging air system can be expressed as a function of wind speed at 10 m above sea level (Eq. 3)

3 $$ {\text{F}}_{{{\text{En}}}} = {2} \times {1}0^{{ - {8}}} {\text{U}}_{{{1}0}}^{{{3}.{74}}} $$

Fluxes F_CN_ were calculated for the equivalent wind speed of each experiment, and then harmonized by using a U_10_ of 9 m s^−1^ and 15 °C seawater temperature to compare fluxes between experiments. We normalized all fluxes to a given seawater temperature so as to isolate the biology impact-only on the SSA fluxes. The temperature dependence was calculated as given for inorganic sea salt by Salter et al. (2015)^48^. This temperature correction induces an average 1.2% increase of the sea spray flux when the flux from Mediterranean seawaters (average 20.4 ± 1.2 °C) is calculated for 15 °C, and a negligible correction to the fluxes was found from arctic and NZ coastal waters (average 13.4 ± 1.9 °C and 17.7 ± 2.2 °C respectively). Using the same set-up as in the present study, Schwier et al. (2017)^24^ measured an average total number flux of 3 × 10^6^ # m^−2^ s^−1^ for a SST of 22 °C in Mediterranean mesocosms seawaters. This would correspond in our parameterization to a nanophytoplankton cell abundance of 1130 cells ml^−1^, which is in the range of nanophytoplankton cell abundances found for the PEACETIME voyage. Our inorganic total number flux are also consistent with number flux measurements from artificial seawater using similar experimental set-ups in Fuentes et al. 2010^41^ and Martensson et al. (2003)^26^. We find a fairly good agreement with these two studies, despite the differences in SSA generation set ups, experimental conditions and flux calculation procedures. Both for the Fuentes (2010)^41^ and Martensson (2003)^26^ parameterizations a flux of 9 × 10^5^ # m^−2^ s^−1^ is calculated for a wind speed of 9 m s^−1^, compared to our inorganic number flux of 2.09 × 10^5^ # m^−2^ s^−1^. Finally, ambient air fluxes of 2 × 10^6^ m^−2^ s^−1^ for a wind speed of 9 m s^−1^ were measured in the Atlantic Ocean using the eddy correlation methods (Geever et al. 2005)^49^, which would correspond to a nanophytoplankton cell abundance of 720 cells ml^−1^ using our parameterization.

### Seawater sampling and characterization

Seawater temperature and salinity were measured during PEACETIME using the onboard thermosalinometer. Chlorophyll-*a* was derived from the particulate absorption spectrum line-height at 676 nm^50^, the relationship was adjusted to PEACETIME chlorophyll-a derived from HPLC (Chl-*a* = 194.41 × line_height^1.131^). POC was estimated from particulate attenuation at 660 nm using an empirical relationship specific to PEACETIME (POC = 1405.1 × c_p_(660) – 52.4) slightly higher than the literature which is likely due to the small dynamic range (1.27 higher on average for the range observed^51^. Both particulate attenuation and absorption of surface water were measured continuously with a WetLabs ACS using a flow-through system similar to the setup described in Slade et al. (2010)^52^. Phytoplankton cells were counted semi-continuously (one sample every hour) using an automated Cytosense flow cytometer (Cytobuoy, NL), connected to the continuous clean pumping system^53,54^. The Cytosense pumped its sample from an isolated chamber of 200 ml filled in less than 30 s. Each sample was driven towards the flow cell by a calibrated peristaltic pump from which volume analyzed were calculated. Each particle in suspension in the sample was then separated one from the other through a laminar flow with a 0.1 µm filtered sea water sheath fluid, and crossed a laser beam (Coherent, 488 nm, 120 mV). The instrument recorded the pulse shapes emitted, resulting in: forward scatter (FWS) and sideward scatter (SWS) as well as red and orange fluorescences (FLR > 652 nm, FLO 552–652 nm respectively) in the size range < 1–800 µm in large and a few mm in length for chain forming cells. Laser scattering at small angles (FWS) was collected by two distinct photodiodes to check for the sample core alignment. Two trigger levels were applied for distinction between highly concentrated picophytoplankton and cyanobacteria groups (trigger level FLR 7.34 mV, sampling at a speed of 4 mm^3^ s^−1^ analyzing 0.65 ± 0.18 cm^3^), and lower concentrated nano- and microphytoplankton (trigger level FLR 14.87 mV, at a speed of 8 mm^3^ s^−1^ analyzing 3.57 ± 0.97 cm^3^). Different sets of 2D projections were plotted in Cytoclus software to manually gate phytoplankton groups. To ensure stability of the flow cytometer, 2 µm red fluorescing polystyrene beads (Polyscience) were regularly analyzed. The use of silica beads (1.0, 2.0 μm, 3.0 μm, 5.0 μm, 7.0 μm in diameter, Bangs Laboratory) for size retrieving estimates from FWS were used to separate picoplankton from nanoplankton clusters.

### Artic waters during MACA

During MACA, phytoplankton (< 20 µm) cell abundances were measured from a subsample of 1.6 mL collected daily for each mesocosm and fixed with glutaraldehyde following the protocol described in Marie et al. 2001 ^55^, then stored at − 80 °C until analysis. The subsample was analysed with FacsCanto II cytometer (3-laser, 8-color (4–2–2), BD Biosciences) equipped with a 20 mW 488 nm coherent sapphire solid state blue laser to evaluate the abundance of cyanobacteria, pico (< 2 µm) and nanophytoplankton (2–20 µm), as described by Pecqueur et al. 2011^56^. During ME3, NanoPhyto were measured by Flow Cytometry using techniques detailed in Safi and Hall (2001)^57^.

### Surface microlayer (SML) sampling and analysis

During PEACETIME, surface microlayer SML sampling was conducted from a zodiac using a 50 × 26 cm silicate glass plate sampler^58^ with an effective sampling surface area of 2600 cm^2^ considering both sides. For sampling, the zodiac was steered 0.5 nautical miles away from the research vessel and into the wind direction to avoid contamination. The glass plate was immersed perpendicular to the sea surface and withdrawn at ~ 17 cm s^−1^. SML samples were removed from the plate using a Teflon wiper^55^ and collected in an acid cleaned and rinsed bottle. Prior to sampling, all equipment was cleaned with acid (10% HCl) and rinsed in MilliQ and copiously rinsed with seawater directly before samples were taken.

The abundance and area of TEP were quantified by microscopy^59^. Depending on the concentration of TEP, 40–50 ml of sample was filtered onto a 0.4 µm Nucleopore membrane (Whatman) and stained with 1 ml Alcian Blue solution (0.2 g l^−1^ w/v) for 3 s. Filters were mounted on Cytoclear slides and stored at − 20 °C until analysis with a Zeiss Axio Scope.A1 (Zeiss). Images were taken with an AxioCam MRc (Zeiss) and analysed with ImageJ ^60^. Filters with MilliQ water served as a blank.

### Ocean color satellite-based estimates of NanoPhyto cellular abundances

Monthly surface NanoPhyto cellular abundances were estimated. Essentially, the method of Uitz et al. (2006)^39^ was applied to the surface chlorophyll *a* concentration derived from the MODIS entire mission monthly composites (9-km spatial resolution). The monthly mixed layer depth data required as input to the method was taken from the climatology of de Boyer Montégut et al. (2004)^61^. This procedure yields estimates of the contribution of nanophytoplankton (comprising nano-eukaryotes and coccolithophores) to total chlorophyll a biomass (in units of mg chlorophyll-*a* m^−3^) within the global ocean, except coastal waters and inland seas. These estimates were then converted into cellular abundances (in units of cells mL^−1^) using an assumed intracellular chlorophyll content of 2.64 × 10^–10^ mg chlorophyll a cell^−1^ for the NanoPhyto group. This was computed as the median of the intracellular chlorophyll content values given in Stramski et al. (2010)^62^ for phytoplankton species within the nanophytoplankton class.

## Supplementary information

Supplementary Information.
