# Supplementary material for: Surface ocean microbiota determine cloud precursors
Source: Sci Rep. 2021 Jan 11;11:281. doi: 10.1038/s41598-020-78097-5 (PMC7801489; doi:10.1038/s41598-020-78097-5)
Supplement: Supplementary file 1 — Supplementary Information. [file 41598_2020_78097_MOESM1_ESM.docx]

Supplementary Materials for

Surface ocean microbiota determine cloud precursors

Karine Sellegri^1^*, Alessia Nicosia^1^, Evelyn Freney^1^, Julia Uitz^2^, Melilotus Thyssen^3^, Gérald Grégori^3^, Anja Engel^4^, Birthe Zäncker^4^, Nils Haëntjens^5^, Sébastien Mas^6^, David Picard^1^, Alexia Saint-Macary^7^, Maija Peltola^1^, Clémence Rose^1^, Jonathan Trueblood^1^, Dominique Lefevre^3^, Barbara D’Anna^8^, Karine Desboeufs^9^, Nicholas Meskhidze^10^, Cécile Guieu^2^ and Cliff S. Law^7^.

^1^ Université Clermont Auvergne, CNRS, LaMP, F-63000 Clermont-Ferrand, France

^2^ Sorbonne Université, CNRS, LOV, F-06230 Villefranche-sur-Mer, France

^3^ Aix-Marseille University, Toulon University, CNRS, IRD, Mediterranean Institute of Oceanography UM110, Marseille 13288, France

^4^ GEOMAR, Helmholtz Centre for Ocean Research Kiel, 24105 Kiel, Germany

^5^ School of Marine Sciences, University of Maine, Orono, ME 04469, USA

^6^ MEDIMEER, UMS3282 OSU OREME (Université de Montpellier, CNRS, IRD), Sète, France

^7^ National Institute of Water and Atmospheric Research (NIWA), Wellington, New Zealand

^7A^ Dept. Marine Sciences, University of Otago, Dunedin, New Zealand

^8^ Université Aix-Marseille, UMR 7673 CNRS, LCE, 13331 Marseille, France

^9^ LISA, CNRS UMR7583, Université Paris Est Créteil (UPEC), Université de Paris, Institut Pierre Simon Laplace (IPSL), Créteil, France

^10^ North Carolina State University, Raleigh, USA

Correspondence to: [K.Sellegri@opgc.cnrs.fr](mailto:xxxxx@xxxx.xxx)

**This PDF file includes:**

Supplementary Text

Table S1

Figs. S1 to S3

Supplementary Text

Nascent sea spray characterization

During PEACETIME, generated SSA size distributions (Figure S1) could be decomposed into 4 modes, centred at 10, 35, 115 and 300 nm and contributing each for 10, 35, 40 and 15% to the total SSA number concentration. Each mode characteristics are given Table S1.

These size distributions are very similar to the ones obtained with the same SSA generation device from coastal Mediterranean seawater at different periods of the year and Chl-a levels *(13,24)*. Average sea spray activation diameters were at 98±10 nm, 49±5 nm and 36±3 nm over the whole measurement period for the tested supersaturations of 0.1%, 0.3% and 0.5% respectively, which is also similar to previous activation diameters found for the Mediterranean seawater during summer (50 nm at 0.4% supersaturation). These activation diameters are representative of low organic content sea spray aerosols, and larger activation diameters were found during pre-bloom conditions (145 nm at 0.1% and 60 nm at 0.4%; *(13)*). In the NZ coastal mesocosm water, activation diameters were measured even higher at 169±16 nm, 67±6 nm and 50±3 nm for corresponding 0.1%, 0.3% and 0.5% supersaturations respectively, indicating larger amounts of organic matter in the SSA generated from these waters. Size distributions of SSA generated from these NZ coastal mesocosm waters showed modes at 36, 105 and 300 nm with respective contributions of 38%, 45% and 15% of the total concentration, which is not very different from size distributions generated from the Mediterranean oligotrophic waters (Table S1). The size distribution of SSA generated from arctic mesocosm seawater show slightly larger contributions from the nucleation and 300 nm modes than for the SSA generated from the Mediterranean seawaters, but the 100 nm mode is still dominating the distribution and modal diameters and sigma are also very similar to the other SSA size distributions (Table S1). This confirms that the organic fraction of SSA does not significantly influence the SSA size distribution shape, as pointed out in the main text.

Seawater property driving the sea spray number production efficiency

The CCN number concentration at 0.3% supersaturation (CCN_0.3_) measured from the SSA generation system along the PEACETIME ship track is shown Figure S2b. Largest concentrations are observed in the south-eastern part of the Mediterranean basin. Chl-a concentrations in the seawater along the PEACETIME ship track is illustrated Fig S2a., showing highest levels on the western part of the Mediterranean basin. The comparison of the two concentration traces clearly shows that Chl-a is an inappropriate tracer for SSA derived CCN production.

Universality of the nanoeukaryotic phytoplankton cell abundance influence on sea spray number and CCN production fluxes

Table 1 is showing the linear coefficients of the fit between the number production flux of SSA larger than 100 nm as a function of the seawater eukaryotic nanophytoplanton, for the Mediterranean and NZ coastal data sets individually, and for the merged Mediterranean, NZ coastal and Arctic databases. The eukaryotic nanophytoplankton cell abundance in the Arctic seawaters were below 5 cells ml^-1^, and could not allow to derive a statistically relevant linear relationship with SSA100. Fitting parameters of the individual data set are close to each other, and to the fitting parameters of the merged data set. The correlation coefficient of the merged datasets is higher than the one obtained for individual databases.

Potential mechanisms for a biologically driven sea spray number flux

The average diurnal variation of the nanophytoplankton cell number abundance and size measured in the Mediterranean seawater during PEACETIME are shown Figure S3a and S3b respectively. A marked diurnal variation of the size of the NanoPhyto is observed, with cells being smaller during nighttime compared to daytime, as a result of cell division during the night, and cell growth during the day. The NanoPhyto cell number abundance is not so clear, but shows an opposite trend, with higher number abundances during night than during late afternoon. This is likely a result of nighttime division modulated either by late afternoon grazing or sea surface layer diurnal dynamics. The average diurnal variation of SSA_100_ (Fig. S3c) and the different organic classes concentrations in the SSA (Fig S3 (d) to (f)) are not marked. Both for NanoPhyto cell abundance and SSA_100_, the spatial variability is higher than the diurnal variability.

|  |  | Diameter (nm) | Amplitude | Sigma | N_SSA100_/N_SSAtot_ |
| --- | --- | --- | --- | --- | --- |
| Arctic mesocosms | Mode Nuc | 8 | 0.007 | 1.5 | 0.58 |
|  | Mode Aitken | 29 | 0.006 | 1.7 |  |
|  | Mode Acc 1 | 93 | 0.0094 | 1.7 |  |
|  | Mode Acc 2 | 290 | 0.007 | 1.5 |  |
| Mediterranean ship | Mode Nuc | 10 | 0.004 | 1.5 | 0.52 |
|  | Mode Aitken | 35 | 0.009 | 1.75 |  |
|  | Mode Acc 1 | 115 | 0.011 | 1.7 |  |
|  | Mode Acc 2 | 300 | 0.004 | 1.4 |  |
| NZ coastal mesocosms | Mode Nuc | NA | NA | NA | 0.51 |
|  | Mode Aitken | 36 | 0.010 | 1.75 |  |
|  | Mode Acc 1 | 105 | 0.011 | 1.7 |  |
|  | Mode Acc2 | 300 | 0.004 | 1.4 |  |

**Table S1.** Parameters of the lognormal decomposition (diameter, amplitude, sigma) of the average normalized size distribution (to the total number emission flux) for each SSA generation experiment. For the NZ coastal mesocosms experiment, size distributions were measured for SSA diameters larger than 20 nm and did not allow to identify a nucleation mode. Average ratio of the SSA number flux which diameter is larger than 100 nm to the SSA total number flux (F_SSA100_/F_SSAtot_) for each experiment.


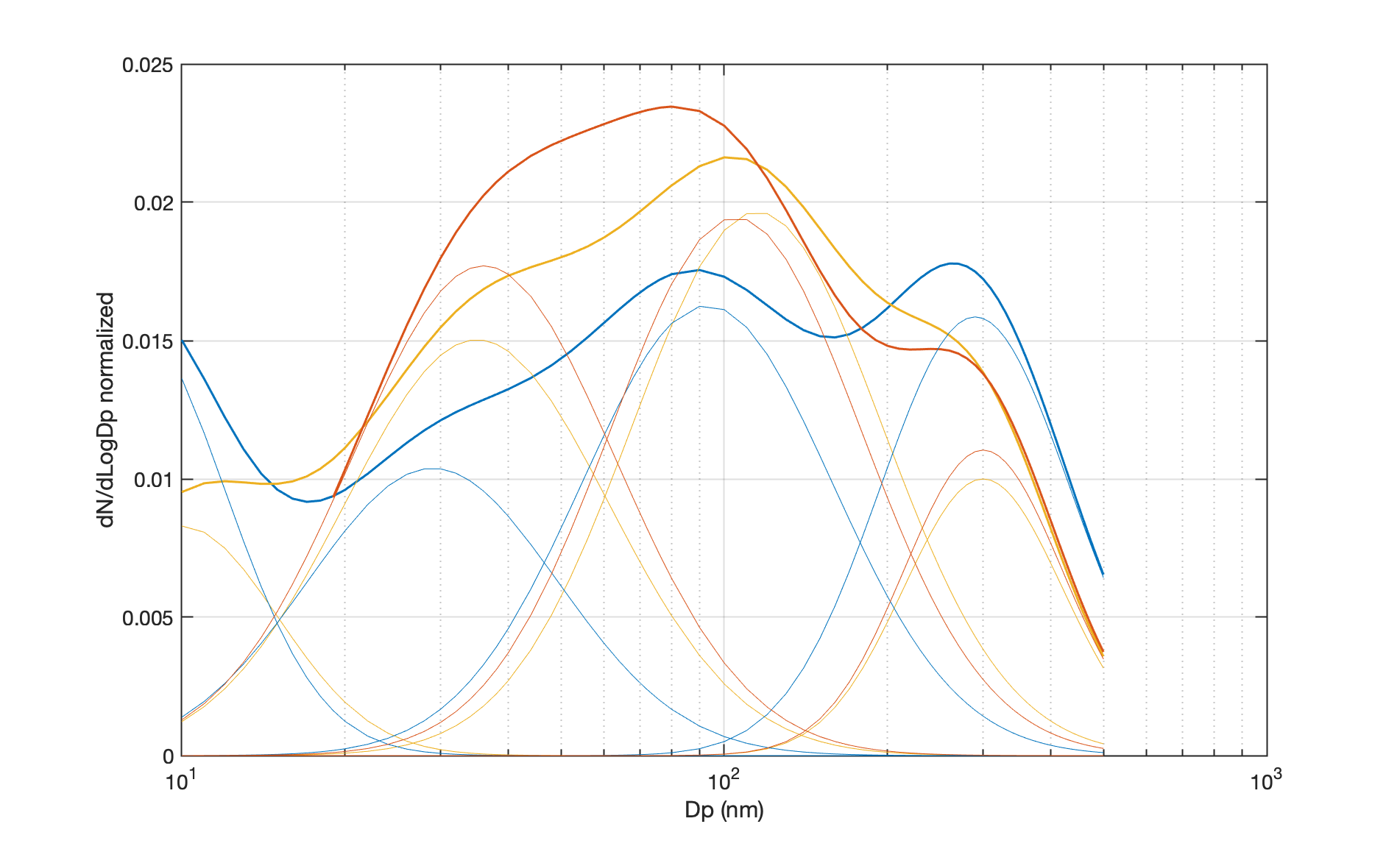


**Fig. S1.** Normalized size distributions in blue: arctic mesocosms, yellow: Mediterranean ship and orange: NZ coastal mesocosms. Bold line are for the full size decomposition and light lines for their mode decomposition.


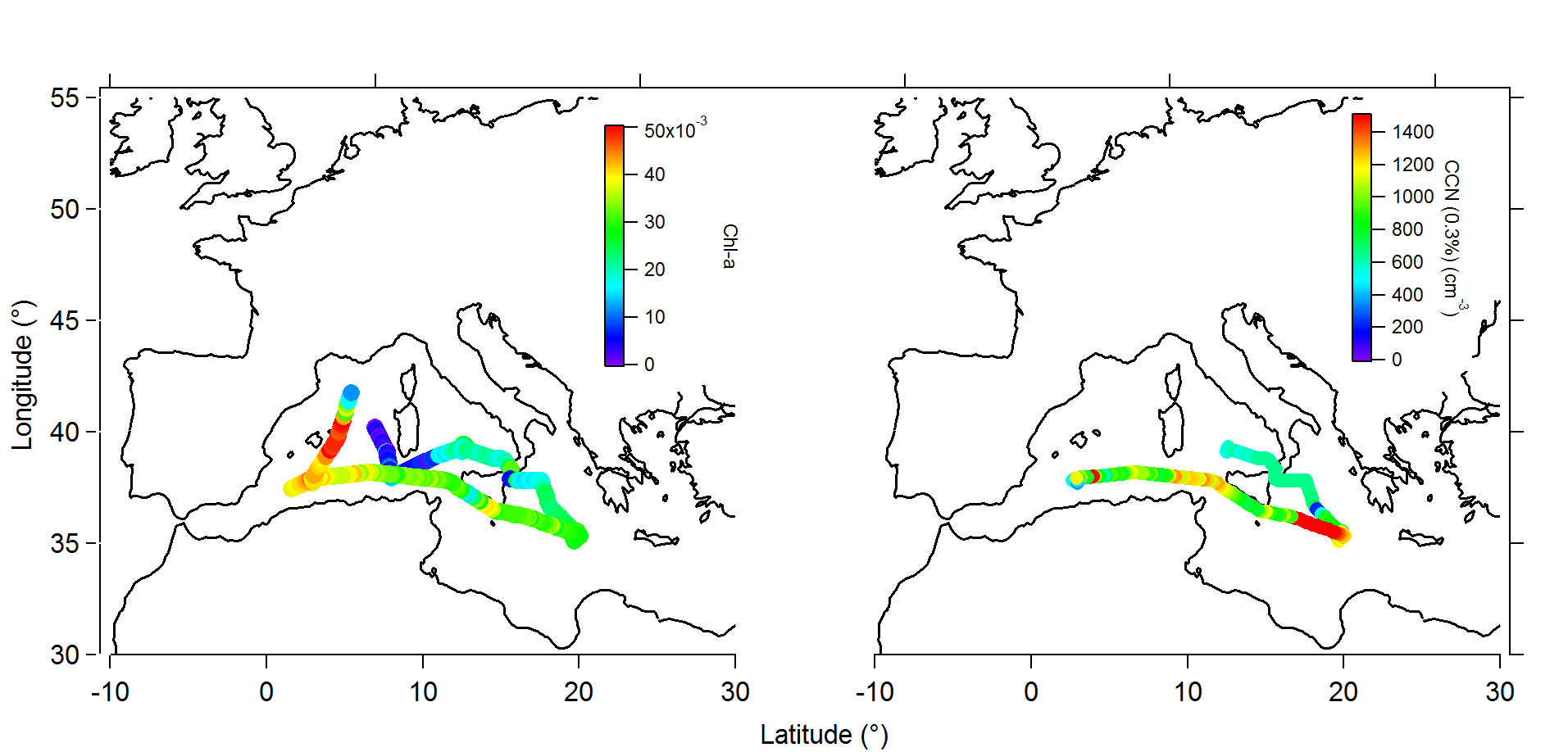
Fig. S2.

Spatial distribution of the (a) surface Chl-a (μg L^-1^) and (b) sea spray originating CCN_0.3_ number  concentration during the PEACETIME cruise in the Mediterranean Sea.


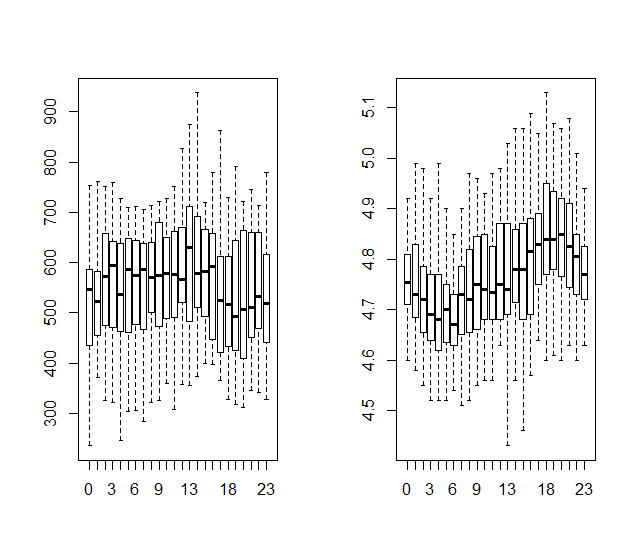


| 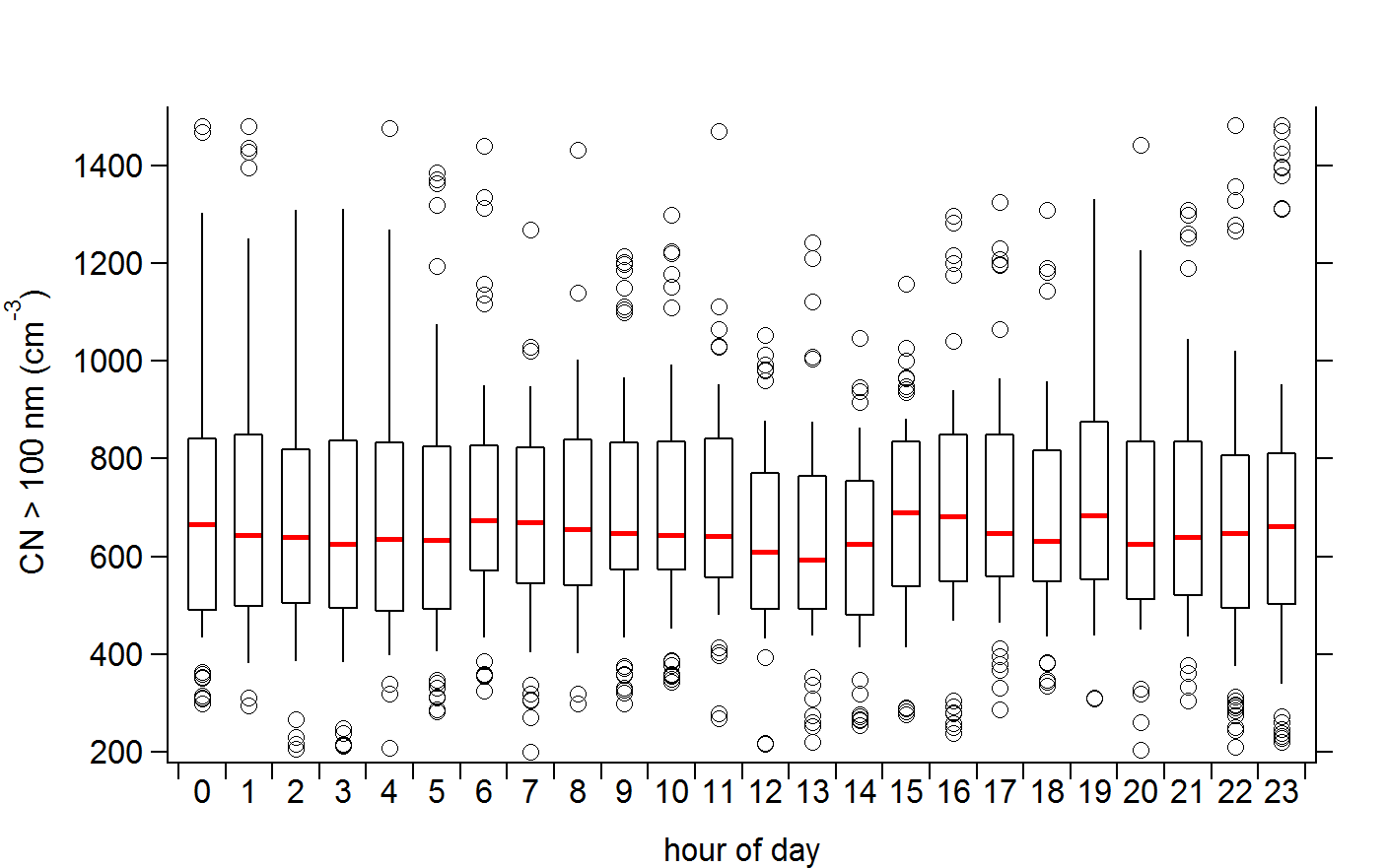 | 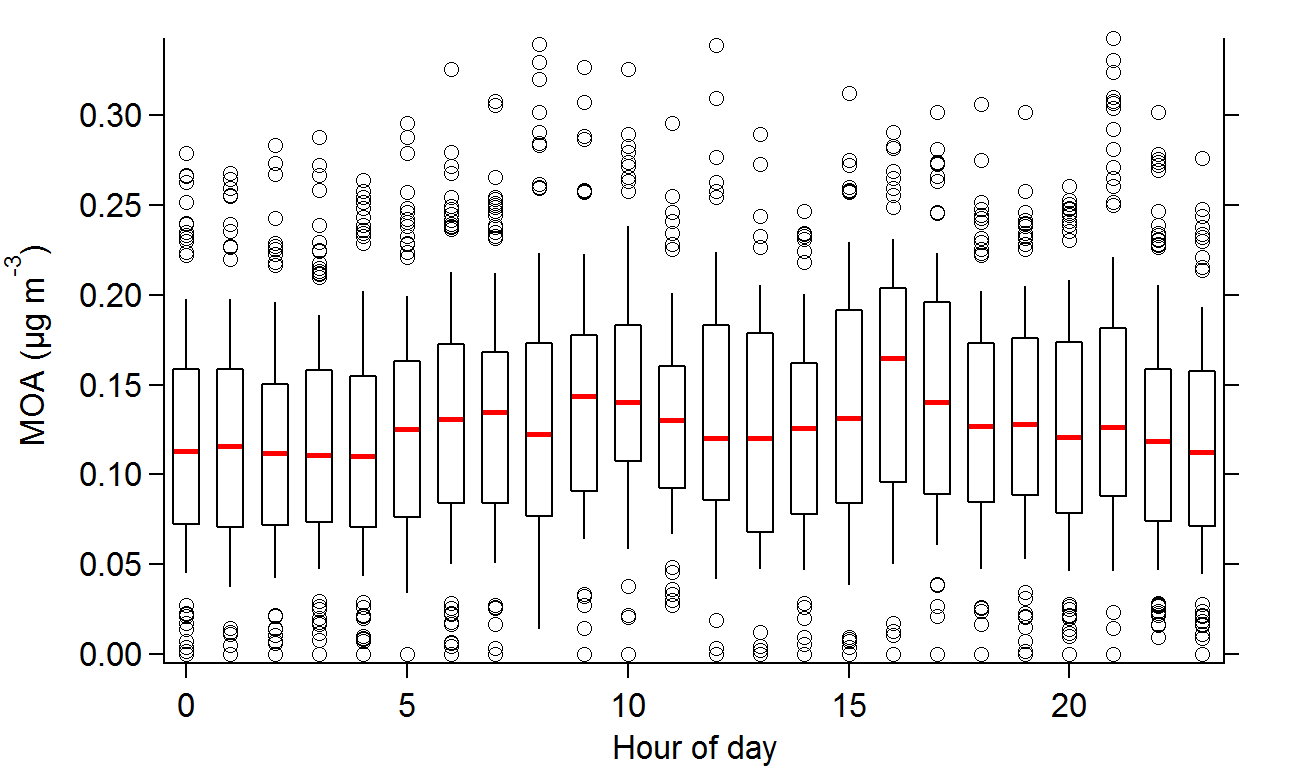 |
| --- | --- |
| 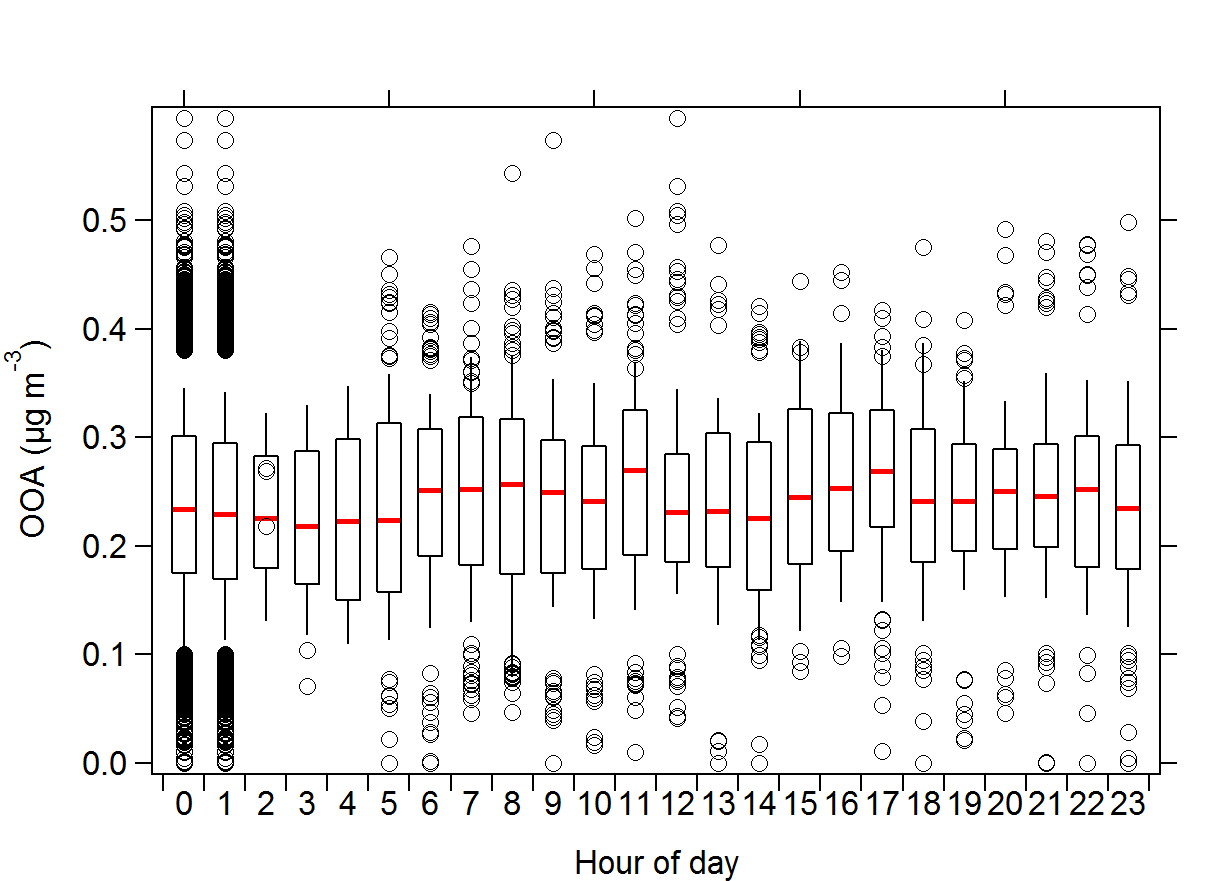 | 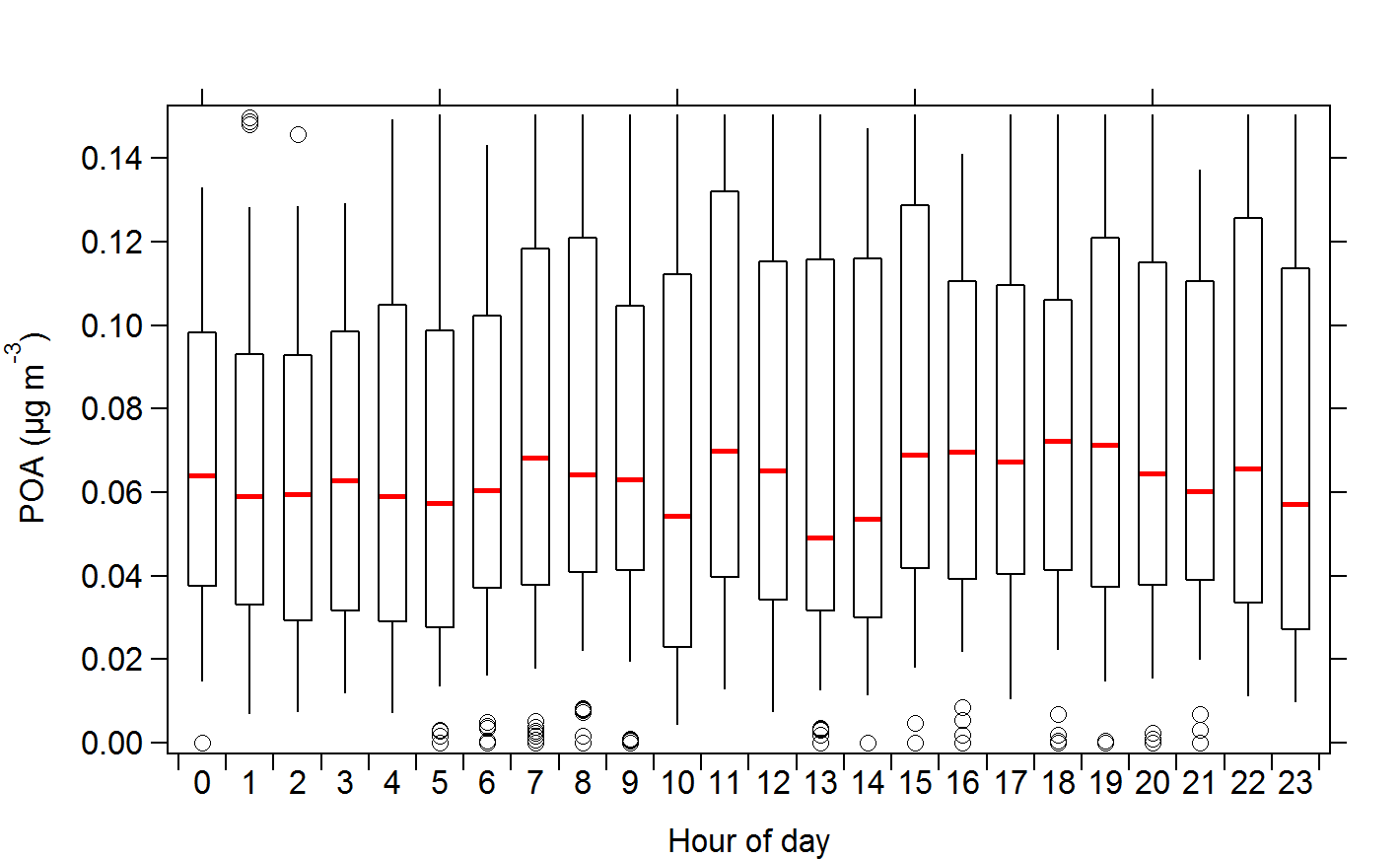 |

Fig. S3.

Mediterranean data set average diurnal variation of (a) nanoeukaryot cell abundance (cells ml^-1^) (b) nanoeukaryots cell size (microns) (c) SSAtot (# cm^-3^) (d) MOA concentrations (µg m^-3^) (e) OOA concentrations (µg m^-3^) and (f) POA concentrations (µg m^-3^).
